# RNAi-Mediated Silencing of Putative Halloween Gene Phantom Affects the Performance of Rice Striped Stem Borer, *Chilo suppressalis*

**DOI:** 10.3390/insects13080731

**Published:** 2022-08-15

**Authors:** Muhammad Faisal Shahzad, Atif Idrees, Ayesha Afzal, Jamshaid Iqbal, Ziyad Abdul Qadir, Azhar Abbas Khan, Ayat Ullah, Jun Li

**Affiliations:** 1 Department of Entomology, Faculty of Agriculture, Gomal University, Dera Ismail Khan 29220, Pakistan; 2 College of Plant Protection, Nanjing Agricultural University, Nanjing 210095, China; 3 Guangdong Key Laboratory of Animal Conservation and Resource Utilization, Guangdong Public Laboratory of Wild Animal Conservation and Utilization, Institute of Zoology, Guangdong Academy of Sciences, Guangzhou 510260, China; 4 Institute of Molecular Biology and Biotechnology, The University of Lahore, 1-Km Defense Road, Lahore 54000, Pakistan; 5 Honeybee Research Institute, National Agricultural Research Centre, Park Road, Islamabad 45500, Pakistan; 6 Department of Entomology and Wildlife Ecology, University of Delaware, Newark, DE 19716, USA; 7 College of Agriculture, Bahadur Sub Campus Layyah, Bahauddin Zakariya University, Multan 31200, Pakistan

**Keywords:** *Cs-phantom* (CYP306A1), cytochrome P450, double-stranded RNA (dsRNA), ecdysteroid biosynthetic pathway, metamorphosis, molting hormone

## Abstract

**Simple Summary:**

Ecdysteroids have a significant role in insect development and reproduction. Ecdysteroidogensis is regulated by a group of genes known as Halloween genes. We provide experimental evidence that *Chilo suppressalis*-*phantom* (*Cs-Phm*) in rice striped stem borer is a Halloween gene phantom and has important role in specific steps in ecdysteroidogensis. Three lines of experiments were done on typical protein domains of *Cs-Phm*, RNAi-mediated knockdown of *Cs-Phm*, and the rescue of larval development in dsRNA-treated groups. The results strongly suggest that *Cs-Phm* plays a critical role in ecdysteroidogensis in rice striped stem borer.

**Abstract:**

The physiological and biochemical characterization of the “Halloween” genes has fundamental importance in the biosynthesis pathway of ecdysteroids. These genes were found to catalyze the final phases of ecdysteroid biosynthesis from dietary cholesterol to the molting hormone 20-hydroxyecdysone. We report the characterization of the *Cs-Phm* in a major insect pest in agriculture, the rice striped stem borer, *Chilo suppressalis* (*C. suppressalis*). A full-length transcript of *Cs-Phm* was amplified with an open reading frame (ORF) of 478 amino acids through 5′ and 3′ RACE. *Cs-Phm* shows five insect-conserved P450 motifs: Helix-C, Helix-I, Helix-K, PERF, and heme-binding motifs. Phylogenetic analysis clearly shows high similarity to Lepidoptera and evolutionary conservation in insects. The relative spatial and temporal transcript profile shows that *Cs-Phm* is highly expressed in the prothoracic glands and appears throughout the larval development, but with low expression at the start of the larval instar. It seems to peak in 3–4 days and decreases again before the larvae molt. Double-stranded RNA (dsRNA) injection of Cs-Phm at the larval stage efficiently knocked down the target gene and decreased its expression level. The dsRNA-treated group showed significantly decreased ecdysteroid titers, which leads to delayed larval development and higher larval mortality. Negative effects of larval development were rescued by treating 20E in the dsRNA-treated group. Thus, in conclusion, our results suggest that *Cs-Phm* is functionally conserved in *C. suppressalis* and encodes functional CYP that contributes to the biogenesis of 20E.

## 1. Introduction

Insect development and growth are coordinated by steroid hormones for developmental transitions [1,2]. Metamorphosis and molting are precisely timed developmental transitions that are strictly regulated by ecdysone in insects [3,4,5]. During larval pupal development, the neuropeptide prothoracicotropic hormone (PTTH) stimulates the synthesis of ecdysone in the prothoracic glands through the activation of mitogen-activated protein kinase (MAPK) and/or phosphoinositide 3-kinase (PI3K) [4,6,7]. Finally, ecdysone is converted to the active hormone 20-hydroxyecdysone (20E) in several peripheral tissues (principally in body fat or the midgut) [8,9,10,11]. 

Steroid hormones′ universal precursor is cholesterol [6]. However, like vertebrates, insects cannot produce it [12]. The biosynthesis of ecdysone includes oxidation and hydroxylation steps during postembryonic development [4]. The initial biosynthetic step of 20-hydroxyecdysone (20E) starts in the endoplasmic reticulum by the conversion of cholesterol to 7-dehydrocholesterol (7dc) by the Rieske-domain protein, Neverland [13]. Subsequently, the oxidation of 7dC to diketol occurs through a series of assumed and unverified “black box” reactions [14]. Then, hydroxylation steps proceed along this pathway. For the hydroxylation reactions, P450 protein is the only catalyzing enzyme [15]. 

Recent studies have shown that Halloween genes encode cytochrome P450 mono-oxygenases involved in the biosynthesis of 20E, such as *spook* (CYP307a1), *phantom* (CYP306a1), *disembodied* (CYP302a1), *shadow* (CYP315a1), and *shade* (CYP314a1) [8,16,17,18,19,20,21,22]. Aside from *spook* (CYP307a1), no paralogs have been identified for the other Halloween genes. In contrast, two additional paralogs exist for *spook*, *spookier* (CYP307a2), and *spookiest* (CYP307B1) [23]. Mutant embryos of the Halloween genes show a similar phenotype characterized by low ecdysteroid titers, dorsal closure, failure of head involution, hindgut abnormality, failure to induce ecdysteroid receptor genes, and lethality [18,19,24]. 

In this study, we cloned the full length of a Halloween gene, *Cs-Phm*, which is speculated to encode an enzyme involved in the biosynthesis of ecdysteroids. The relative spatio-temporal expression patterns were analyzed using quantitative real time PCR (qRT-PCR). We also investigated whether dsRNA-mediated knockdown of *Cs-Phm* induces delayed larval development and mortality. To validate our results, topical application of 20E was used to rescue the negative effects of dsRNA-injected insects. The results of this study provide basic information on *Cs-Phm* and its functional importance in ecdysteroidogenesis in the rice striped stem borer, *C. suppressalis*.

## 2. Materials and Methods

### 2.1. Insects

Individuals of *C. suppressalis* were collected from rice fields in Nanjing (32.00° N, 118.50° E), Jiangsu province, China, and cultured routinely in an insectary. The collected strain has been reared in an insectary on rice seedlings at 25 ± 3 °C and a light:dark ratio of 16:8 with approximately 80% relative humidity. 

### 2.2. Total RNA Isolation and cDNA Synthesis

Total RNA was extracted from 4th-instar larvae (~3 pooled larvae) using TRIzol reagent (Invitrogen, Grand Island, NY, USA) and an RNeasy micro kit (Qiagen Inc., Valencia, CA, USA) according to the manufacturer′s instructions. Genomic DNA was removed from RNA samples with RNase-free DNase I using the protocol of the DNA-free kit (Roche Diagnostics, Mannheim, Germany). The RNA purity and concentration were measured with a NanoDrop^TM^ ND1000 spectrophotometer (NanoDrop Technologies, Rockland, DE, USA). cDNA was synthesized using a PrimeScript^TM^ Ist Strand cDNA Synthesis Kit (TaKaRa Bio., Dalian, China) from 1 μg of total RNA by following the manufacturer′s instructions. It was then used as a template for PCR. 

### 2.3. Amplification of cDNA Fragment

The annotation-based *Cs-Phm* transcript was searched for in the *C. suppressalis* draft genome [25]. Based on the transcript, primers were designed using the software Primer Premier version 5.0 [26] and are listed in Table 1. PCR was performed according to the following program: 94 °C for 3 min followed by 30 cycles at 94 °C for 30 s, 55 °C for 40 s, and 2 min at 72 °C, with a final elongation at 72 °C for 10 min. Each 50 μL PCR reaction mixture contained 2 μL of cDNA template, 0.5 μL of LA Taq polymerase, 5 μL of MgCl_2_ (25 mM), 5 μL of 10 × LA Taq buffer, 8 μL of dNTP mixture (2.5 mM/each), 1 μL of sense primers, 1 μL of antisense primers (10 μM) (TaKaRa Bio, Dalian, China), and 27.5 μL of double-distilled H_2_O. The PCR product was cleaned using a Wizard DNA Gel Extraction Kit by following the manufacturer′s protocol (Promega, Madison, WI, USA) and then sequenced. 

### 2.4. Rapid Amplification of cDNA Ends (3′ RACE and 5′ RACE)

To amplify the full-length cDNA of *Cs-Phm*, we carried out rapid amplification of cDNA ends (RACE): 5′ and 3′ RACE-ready cDNA were prepared using a SMARTer RACE cDNA amplification kit (Takara Bio, Dalian, China) from the total RNA that was extracted from whole bodies of 4th-instar larvae (~3 pooled larvae). Gene-specific primers (GSP) and nested gene-specific primers were designed by partial sequences using Primer Premier 5.0 to amplify the 5′ and 3′ ends. The first PCR was performed with GSP and universal primer mix according to the following program: incubation for 3 min at 94 °C, 5 cycles at 94 °C for 30 s, 72 °C for 3 min; 5 cycles for 30 s at 94 °C, 70 °C for 30 s, 3 min at 72 °C; and 25 cycles at 94 °C for 30 s, 68 °C for 30 s, and 72 °C for 3 min, with final elongation at 72 °C for 10 min. In the nested gene-specific primer PCR, the first round of PCR product was diluted by 100-fold and then used as a template with nested gene-specific primer and nested universal primer mix. PCR was performed according to the following program: incubation for 3 min at 94 °C, followed by 20 cycles at 94 °C for 30 s, 68 °C for 30 s, and 72 °C for 3 min. The final extension was 72 °C for 10 min. 

The amplified PCR products were separated by 1.2% agarose gel, cleaned using a Wizard DNA Gel Extraction Kit, and cloned into easy vector pGEM-T (Promega, Madison, WI, USA). Several of the longest clones were sequenced from both directions with the help of an automated sequencer (Model-3730, Applied Biosystems, Foster City, CA, USA). After amplifying the full-length cDNA of *Cs-Phm*, we designed forward and reverse primers using Primer Premier 5.0 software [26] to confirm the complete open reading frame (Table 1). The DNAStar program (http://www.dnastar.com accessed on 12 April 2019) was used to predict the open reading frame. The resulting *Cs-Phm* sequence was submitted to GenBank^®^ (KF701127).

### 2.5. Phylogenetic Analysis

The following phantom amino acid sequences were downloaded from the NCBI GenBank^®^ database (http://www.ncbi.nlm.nih.gov accessed on 12 April 2019): *Spodoptera littoralis* (ACM45975); Ha—*Helicoverpa armigera* (AID54855); Mb—*Mamestra brassicae* (BAN66311); Ms—*Manduca sexta* (ABC96068); Bm—*Bombyx mori* (BAM73853); Dp—*Danaus plexippus* (EHJ76071); Zn—*Zootermopsis nevadensis* (KDR21920); Ae—*Aedes aegypti* (EAT43717); Ad—*Anopheles darling* (ENT60005); Dm—*Drosophila melanogaster* (NP573319); and Tc—*Tribolium castaneum* (XP968477). The neighbor-joining technique was used to build a phylogenetic tree with 1000 bootstrap replicates using MEGA version 5.0 with the complete deletion of gaps and a Poisson model [27].

**Table 1:** Primers used for validation of *Cs-Phantom*, 5′ and 3′ RACE, and quantitative real-time PCR.

Primer	Sequence (5′ to 3′)	Amplicon Size (bp)
Primers used in RT-PCR		
Phm-RT-F	AGCAACCTCATTTGACCCTA	483
Phm-RT-R	GGCACCCTTCTTCTTGGA	
Primers used in 5′-RACE		
Phm-5GSP	CACCCTTCTTCTTGGATCTGACGAAACC	
Phm-5NGSP	CAGCACATATGATACCATTGCCACGC	
Primers used in 3′-RACE		
Phm-3GSP	ACTGGAAAAACGCATCGCTGCTGGC	
Phm-3NGSP	GGTTTCGTCAGATCCAAGAAGAAGGGTG	
Primers used in PCR for End to End		
Phm-F	ATGGGGAGTCGCTGGTCA	1717
Phm-R	CCCGCACCGACGTGGTAT	
Primers used for synthesizing the dsRNAs Phm-dsRNA-F Phm-dsRNA-R GFP-dsRNA-F GFP-dsRNA-R Primers used in qRT-PCR	taatacgactcactataggTGACACCAAAAGGAGCGGAA taatacgactcactataggGCCCACTGGAGAGGTATCAC taatacgactcactataggTCACGGATACAACCTCTTT taatacgactcactataggAGTTCAGCGTGTCCG	544 414
Phm-qF	GCCAGGTGATAGGTTGTGTT	204
Phm-qR	ATGAGGTTGCTTGGGATCTATG	

### 2.6. Quantitative Reverse Transcription Analysis

Whole-body total RNA was isolated from the third- to sixth-instar larvae and pupae to determine the *Cs-phm* expression pattern at different developmental stages. Epidermis tissues, prothoracic glands, midgut, Malpighian tubules, and body fat RNA was isolated from fourth-instar larvae to measure *Cs-phm′s* spatial expression using TRIzol reagent according to the manufacturer′s directions (Invitrogen, Grand Island, NY, USA). Pooled samples of three animals were used for developmental expression, and pooled samples of 10–20 animals were used to collect samples for tissue expression. 

Genomic DNA was removed from RNA samples with RNase-free DNase I using the protocol of the DNA-free kit (Roche Diagnostics, Mannheim, Germany). qRT-PCR analysis was performed using three biological replicates for each sample. We used an online program, PrimerQuest (http://www.idtdna.com/scitools/applications/primerquest/ accessed on 12 April 2019), to design qRT-PCR primers (Table 1). In our previous study, we found that *Actin-A1* was the most stable housekeeping gene in *C. suppressalis*, so it was also used in this study [28]. 

Real-Time PCR ABI 7500 System (Takara Bio, Dalian, China) was performed with 96-well plates using SYBR PremixEx Taq^TM^ (Takara Bio, China) in technical triplicate reactions by following the manufacturer’s protocol. The reaction mixture had a final volume of 20 μL and contained 2 μL of cDNA template, 1 μL of forward primer (10 μmol/L), 1 μL of reverse primer (10 μmol/L), 10 μL of SYBR Premix Ex Taq (Takara Bio), and 0.4 μL of Rox Reference Dye (50×). A non-template negative control and a reverse-transcription negative control (without reverse transcriptase) were included for each primer set to detect contamination of primer–dimer and genomic DNA in the reaction. 

The qRT-PCR program included pre-denaturing at 95 °C for 30 s and then 40 cycles at 95 °C for 5 s and 60 °C for 34 s. After amplification, the samples were heated at 95 °C for 15 s to determine the melting curves, followed by cooling down for 1 min at 60 °C, and finally heating for 15 s at 95 °C. All data were analyzed using the 2^−ΔΔCt^ method [29]. For ΔΔCT calculation to be valid, the amplification efficiencies of target and reference gene must be approximately equal. A cDNA preparation was diluted by 100-fold to investigate how ΔCT (CT,_Cs-Phm_-CT,_Actin-A1_) varies with the template dilution. A plot of the log CDNA dilution versus ΔCT was made. The absolute value of slope was 0.0472, which is close to 0. This calculation clearly showed that efficiencies of target and reference gene were similar; therefore, ΔΔCT calculation for the relative quantification of *Cs-phm* may be used [30].

### 2.7. dsRNA Preparation and Microinjection

A T7 RibomaxTM Express RNAi System (Promega) was used to prepare double-strand RNA (dsRNA), and pEASY-T3 vector (TransGen Biotech, Beijing, China) was used to clone the cDNA of green fluorescent protein (GFP) and *Cs-Phm*. T7 promoter sites were added by PCR using diluted plasmid templates and modified GSPs (Table 1). Wizard HSV Gel (Promega) was used to purify PCR products for templates, and dsRNA was synthesized by the T7 RibomaxTM Express RNAi System using the manufacturer′s protocol. Two complementary RNA transcripts were prepared and hybridized to synthesize dsRNA. The products were treated with RNase and DNase I with incubation at 37 °C for at least 30 min to remove any single-stranded RNA and DNA. Isopropanol was used to precipitate the synthesized dsRNA, which was then resuspended in nuclease-free water. 

The dsRNA quantity was analyzed at 260 nm using a NanoDrop^TM^ ND1000 spectrophotometer (Nano-Drop Technologies, Rockland, DE, USA). The dsRNA product aliquots were measured with a 1.2% *w/v* agarose gel run in TAE buffer (40 mmol/L Tris acetate, 2 mmol/L Na2EDTA·2H_2_O). Resulting bands were visualized by ethidium bromide staining. The required dsRNA concentration of 2400 ng/μL was diluted by adding nuclease-free water. 

On day 2, fourth-instar larvae were injected with 2 μL of dsRNA between the third and fourth segments using an InjectMan NI-2 microinjection system (Eppendorf, Germany) in two groups. dsGFP injection was used as a negative control in one group, and ds*Cs-Phm* injection was used for treatment in the other group. Needles were prepared using glass capillaries (outer diameter 1.00 mm, and inner diameter 0.50 mm) and a micropipette puller (Model P-87, Sutter Instruments Company, Novato, CA, USA). To avoid dsRNA leakage, needles were kept in the insect body for at least 30 s at the injection point. 

### 2.8. Cs-Phm Relative Expression Level in dsRNA-Treated Insects

Phenotypic observations were recorded in the first group, and mRNA abundance after dsRNA injection was estimated in the second group (40 individuals for each treatment). We collected samples at 1, 2, 3, and 4 days after dsRNA injection to estimate mRNA abundance. The mRNA abundance of *Cs-Phm* in the dsRNA-treated group was normalized with the data at the same time point with the negative control. Independent triplicate biological replications were used in all experiments. 

Isolation of total RNA and cDNA preparation were the same as explained above. We used the *Actin-A1* housekeeping gene (ChiloDB Accession Number = CSUOGS101387-TA) as a reference gene in all experiments [25,28]. An ABI 7500 Real-Time PCR System (Applied Biosystems) was used according to the instructions of the manufacturers to measure the mRNA abundance. The molting and mortality percentage were calculated at 3 days post-injection. The data were analyzed by analysis of variance (ANOVA) using SPSS for Windows (SPSS version 24, Chicago, IL, USA) and are presented as the means ± SE. All graphs were prepared by using Graph Pad Prism 5.

### 2.9. 20-Hydroxyecdysone Titer Measurement

Samples were collected at 72 h after dsRNA injection. Each sample contained 3 larvae and was repeated in biological triplicate. The whole larval bodies were homogenized with 70% methanol and incubated at 60 °C for 10 min, followed by centrifugation at 10,000× *g* for 10 min. 70% methanol was used to extract the resulting pellets, and supernatants were dried under low pressure. Hexane and 70% methanol were mixed with dried residues to remove apolar lipids [31]. A standard procedure to remove the hexane phase was adopted. Methanol was desiccated, and the sample redissolved in 300 μL of 70% methanol. 

High-performance liquid chromatography (HPLC) with an XDB-C18 column (4.60 × 250 mm, 5 μm, Agilent Technologies, Santa Clara, CA, USA) was used to measure ecdysteroids, and 20-E was separated using a binary gradient elution. Mobile phase A was water containing 0.1% formic acid, and mobile phase B was acetonitrile containing 0.1% formic acid. The analysis was done using a described method [32]. For this, 20E (Sigma, St. Louis, MO, USA) was used as a standard for 20-hydroxyecdysone measurement.

### 2.10. Rescue Analysis

We used 20E (Sigma, St. Louis, MO, USA) to rescue the ds*Cs-Phm* phenotype. Topical application of a 0.25 μL aliquot of acetone with or without 20E (1.00 mg/mL) on the thoracic surface of the larvae was used on ds*Cs-Phm*-injected larvae at 24 h post-injection. Each group had 40 individuals with three biological replicates.

### 2.11. Statistical Analysis

The data are presented as the means ± SE, analyzed by ANOVA, and compared by A Tucky–Kramer test using SPSS for Windows (SPSS Inc., Chicago, IL, USA).

## 3. Results 

### 3.1. Molecular Cloning and Sequence Analysis of Cs-Phm

**Figure 1:**
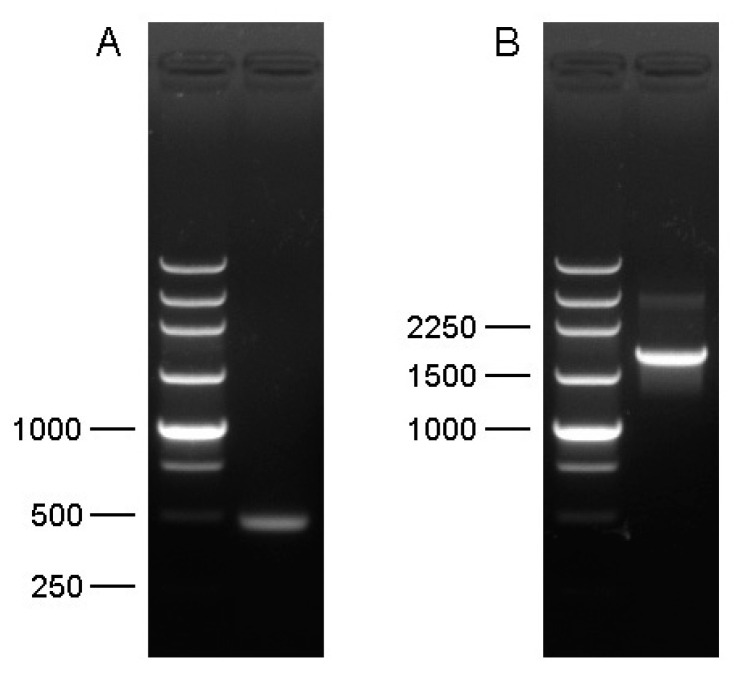
Elelctrophoretic analysis of (**A**) partial fragment and (**B**) end-to-end RT-PCR for full-length transcript of *Cs-Phm*.

**Figure 2:**
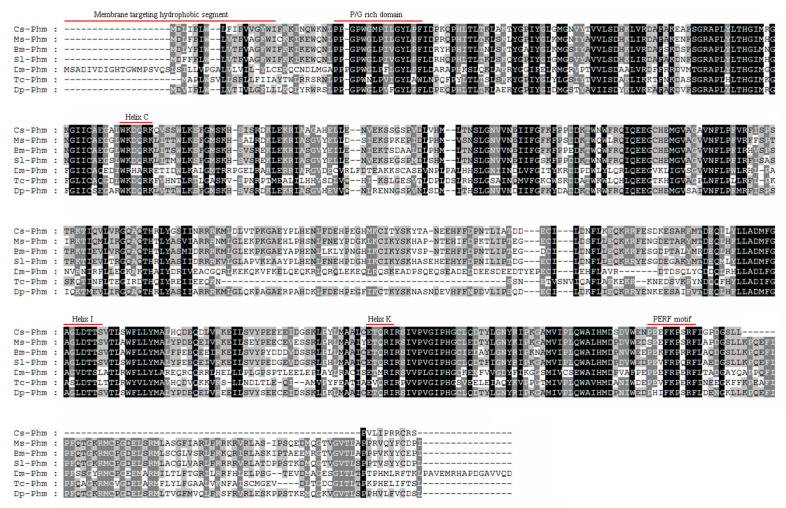
Alignment of CYP306A1 sequences originating from *C. suppressalis* (KF701127), Ms—*Manduca sexta* (ABC96068), Bm—*Bombyx mori* (BAM73853), Sl—*Spodoptera littoralis* (ACM45975), Dm—*Drosophila melanogaster* (NP573319), Tc—*Tribolium castaneum* (XP968477) and Dp—*Danaus plexippus* (EHJ76071). Amino acids shaded in black, dark grey, and light grey demonstrated 100%, >80%, and >60% conservation, respectively.

A full-length *Cs-Phm* sequence of a 2045 bp ORF encoding a protein of 478 amino acids was obtained through 5′ and 3′ RACE (Figure 1). To assign the specific name, we submitted it to the Nomenclature Committee for Standardized Cytochrome P450 [33]. Moreover, the resulting full-length sequence was submitted to the National Centre for Biotechnology (GenBank, NCBI) (accession number KF701127). Protein alignment of *Cs-Phm* with other insects′ known phantom proteins showed similar cytochrome P450 conserved motifs (Figure 2). 

Insects Cytochrome P450 has five conserved P450 motifs: WxxxR (Helix-C), GxE.DTT/S (Helix-I) ExxR (Helix-K), PxxFxPxRF (PERF), and PFxxGxRxCxG/A (Heme-binding), where x means any amino acid [34]. We observed that the first motif WxxxR is located in helix-C, which is believed to form a charge pair with the propionate of the heme by arginine. The second conserved motif, AGxxT, is located in helix-I, which is used to transfer a proton groove on the distal side of heme. The third conserved P450 motif is located in helix-K (ExxR), which stabilizes the enzyme through a set of salt bridge interactions. 

**Figure 3:**
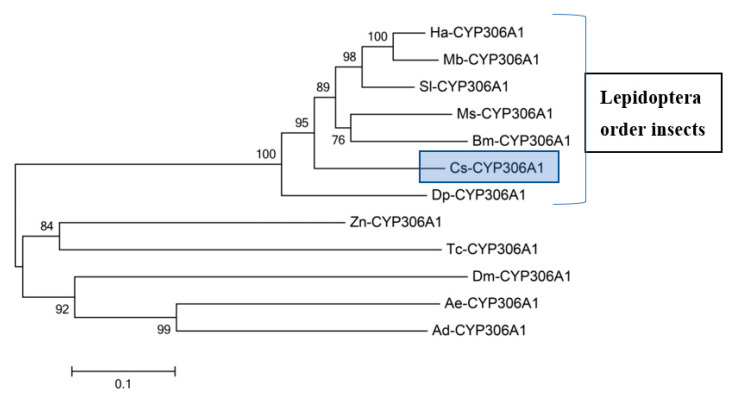
Phylogenetic tree of a Halloween gene *Cs-Phm*. The tree was generated using MEGA version 5.0 with a bootstrap value of 1000 trials for each branch position based on the whole amino acid sequences by neighbor-joining method excluding the gap position. Phantom-like proteins originate from Sl—*Spodoptera littoralis* (ACM45975), Ha—*Helicoverpa armigera* (AID54855), Mb—*Mamestra brassicae* (BAN66311), Ms—*Manduca sexta* (ABC96068), Bm—*Bombyx mori* (BAM73853), Dp—*Danaus plexippus* (EHJ76071), Zn—*Zootermopsis nevadensis* (KDR21920), Ae—*Aedes aegypti* (EAT43717), Ad—*Anopheles darling* (ENT60005), Dm—*Drosophila melanogaster* (NP573319) and Tc—*Tribolium castaneum* (XP968477). The percentiles of bootstrap values (1000 replicates) are indicated. The amino acid divergence is shown in the scale bar.

The PERF (PxxFxPxRF) motif is the fourth domain and is present in the sequence in an aromatic region. Moreover, PFxxGxRxCxG/A (heme-binding domain) serves as a ligand to heme iron by conserved cysteine [34]. The phylogenetic tree was constructed with the neighbor joining method in MEGA version 5.0 and CLUSTALX, which clearly shows high similarity (≥73%) and identity (≥63%) of *Cs-Phm* with insects belonging to the Lepidoptera order compared to others (Figure 3). 

### 3.2. Spatial Transcript Profiles

**Figure 4:**
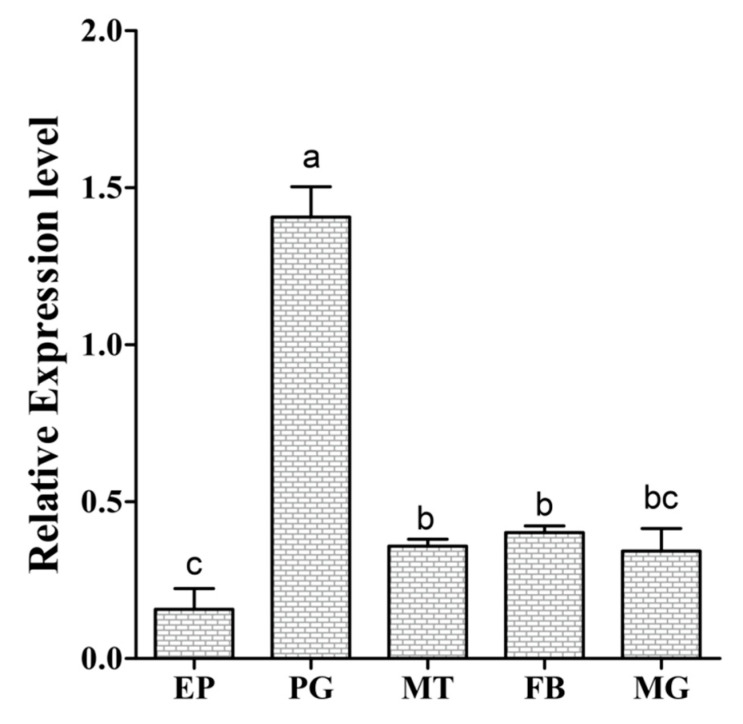
The relative *Cs-Phm* transcript level measured in various larval tissues using qRT-PCR. The larval tissues (EP—epidermis; PG—prothoracic glands; MT—Malpighian tubules; FB—body fat; and MG—midgut) were dissected from 4th day of 5th-instar larvae. The columns indicate average values, and vertical bars represent SE. Different letters indicate significant differences at *p* value < 0.05.

qRT-PCR was used to understand the functional importance of *Cs-Phm* in the selected tissues. The relative spatial transcript profile from the epidermis tissues, prothoracic glands, midgut, Malpighian tubules, and body fat showed that *Cs-phm* is mainly expressed in the prothoracic glands (Figure 4). A low transcript level of *Cs-phm* also appeared in the midgut, Malpighian tubules, and body fat.

### 3.3. Temporal Transcript Profiles

**Figure 5:**
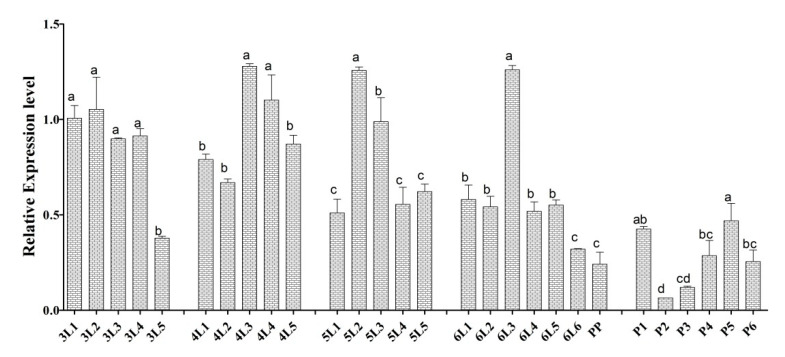
The relative *Cs-phm* transcript level measured throughout 3rd- to 6th-instar larvae and pupae after 24 h intervals using qRT-PCR. The columns indicate average values, and vertical bars represent SE. Different letters indicate significant differences at *p* value < 0.05.

The *Cs-phm* relative transcript was measured throughout 3th- to 6th-instar larval development and pupal development to determine the expression pattern at different developmental stages (3rd-, 4th-, 5th-, and 6th-instar larvae and pupae with average times of 5, 5, 5, 7, and 6 days at our insectary temperature, respectively). *Cs-Phm* was present throughout the larval development, but at the beginning, it had low expression during the larval instar and seemed to peak on days 3–4. It decreased again before the larvae molt. However, in the 3rd-instar larvae, the *Cs-phm* level was relatively high throughout the instar. A similar relative expression trend was observed throughout the third to sixth larval instars. In contrast to larvae, high expression was observed during the first day of pupal development, followed by a significant decrease and then a peak at day 4 before pupal transition (Figure 5). 

### 3.4. Effects of dsRNA Injection

**Figure 6:**
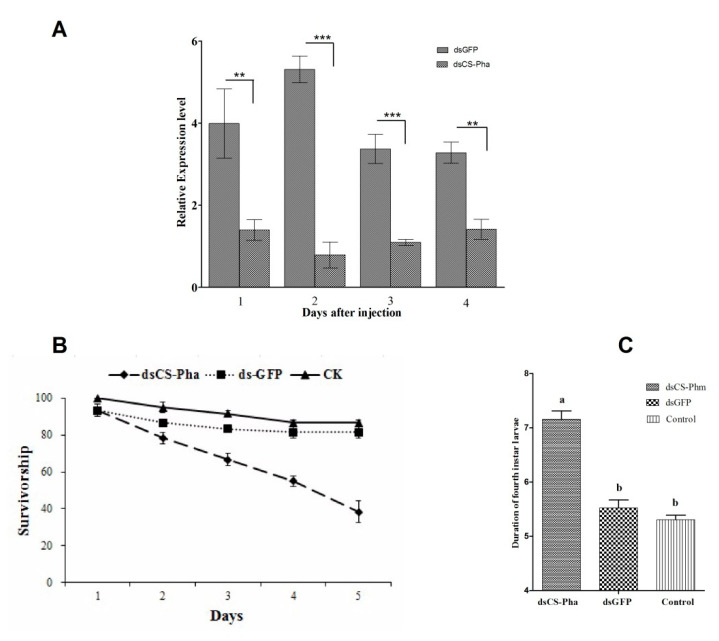
Effects of ds*Cs-Phm* injection on the (**A**) relative expression of *Cs-phm* (**B**) larval survival (**C**) and larval development of rice striped stem borer, *C. suppressalis.* ds*Cs-Phm* was injected on day 2 in 4th-instar larvae. The relative transcript level of *Cs-Phm* was measured after 1, 2, 3, and 4 days after ds*Cs-Phm* injection. Each sample collected for *Cs-Phm* relative expression measurement contained two larvae. The larval survival and larval development in 4th-instar larvae was calculated from three batches with 40 individuals in each batch. The columns indicate average values, and vertical bars represent SE. ** and *** indicate statistical differences between treatments at *p* < 0.01 and *p* < 0.001, respectively. Different letters in vertical bars represent significant differences at *p* value < 0.05.

The efficacy of ds*Cs-Phm* injection was robust (Figure 6). The relative expression level of *Cs-Phm* in ds*Cs-Phm*-injected insects after 1, 2, 3, and 4 days was reduced by 64.91%, 85.11%, 67.56%, and 56.81%, respectively, compared to insects injected with dsGFP (Figure 6A). Mortality was calculated in fourth-instar larvae injected with ds*Cs-Phm* and compared to the control group. We observed 61.67% mortality in the larvae injected with ds*Cs-Phm*, while only 13.33% and 18.33% mortality was observed in the control and the group injected with dsGFP, respectively (Figure 6B). Larval development was also observed in the treated and control group. Larvae that were injected with ds*Cs-Phm* showed significantly delayed larval development compared to the control group. The average fourth-instar larval stage was longer in the ds*Cs-Phm* RNAi group than the controls, spanning ~ 7d rather than ~5 d (Figure 6C). The data clearly showed that larval development was significantly delayed in larvae injected with ds*Cs-Phm*. 

### 3.5. Ecdysteroid Titer Measurement

**Figure 7:**
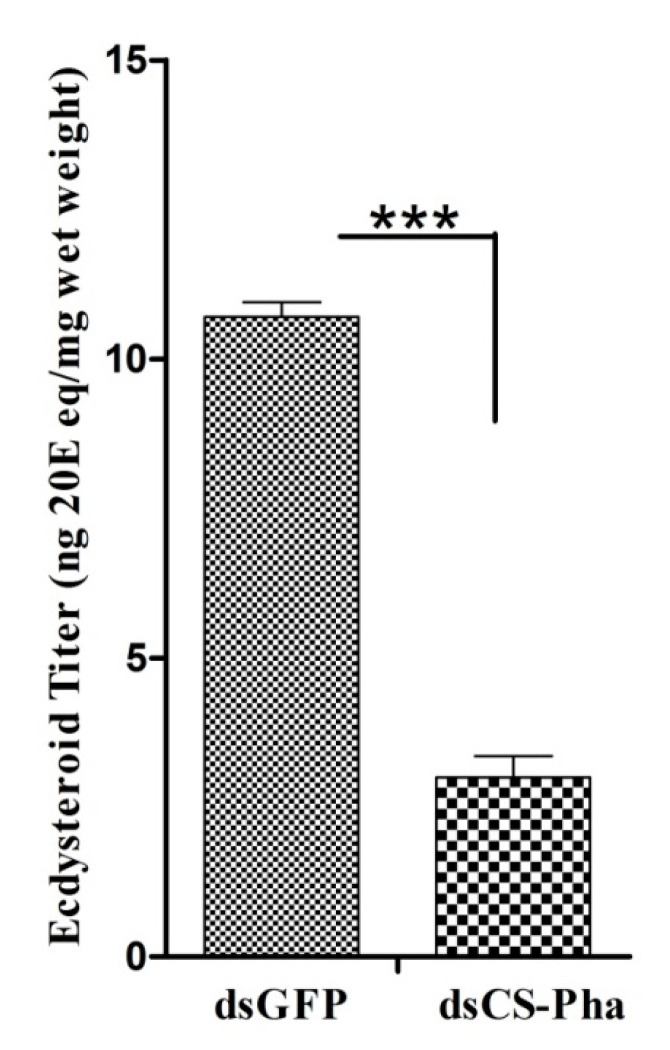
Ecdysteroid titers of dsGFP and ds*Cs-Phm*-injected larvae measured with HPLC. The columns indicate average values, and vertical bars represent SE. *** indicates statistical differences between treatments at d *p* < 0.001.

Larvae injected with ds*Cs-Phm* showed significantly decreased ecdysteroid levels compared to the dsGFP-injected larvae in the control group (Figure 7). The highly decreased ecdysteroid titer clearly indicates the involvement of *Cs-Phm* in ecdysteroid biosynthesis.

### 3.6. Rescue Experiment

**Figure 8:**
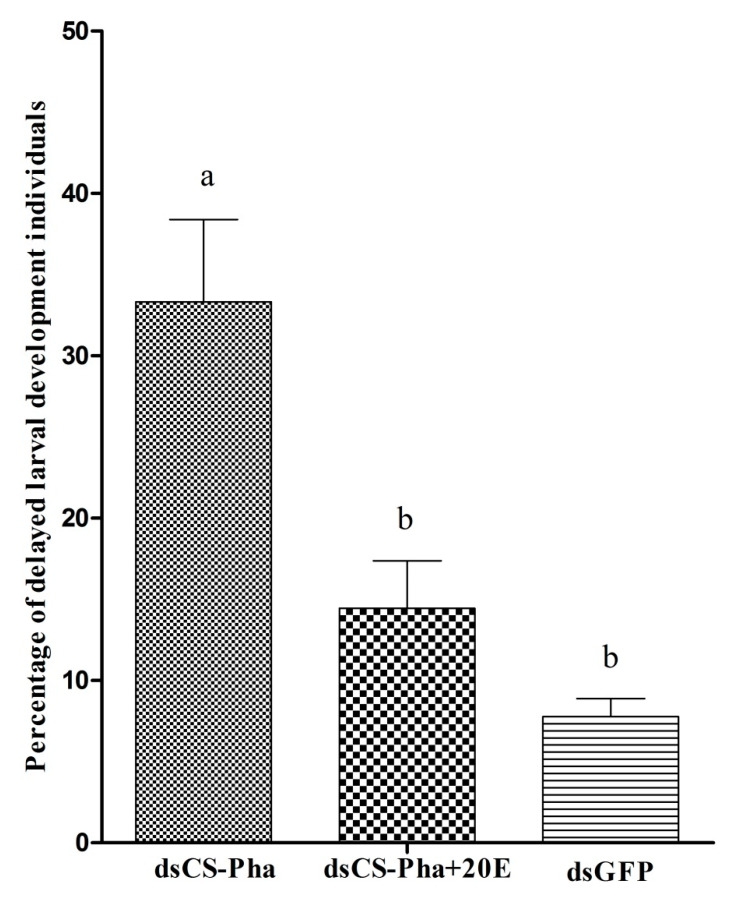
Delayed larval individuals subjected to ds*Cs-Phm*, ds*Cs-Phm* +20E, and dsGFP. The columns indicate average values, and vertical bars represent SE. Letters above the bars indicate statistical differences between treatments at *p* < 0.05.

Topical application of 20E (Sigma, St. Louis, MO, USA) at 24 h post-injection on the thoracic surface of larvae was used to address whether delayed larval molting in ds*Cs-Phm* larvae was due to decreased ecdysteroid titer. It was observed that larvae injected with ds*Cs-Phm* had 33.32% delayed larval molting compared to the control group with dsGFP injection, which had only 7.78% delayed larval molting. The percentage of delayed larval molting was completely rescued with topical application of 20E (Sigma, St. Louis, MO, USA) at 24 h post-injection at 14.44%, which is similar to the control group with dsGFP injection (Figure 8).

## 4. Discussion

As metamorphosis and molting are fundamental phenomena during arthropod development, the Halloween genes in insects are well conserved [8,9,35,36,37,38,39], as in other arthropods [40,41]. In this report, we demonstrated the characterization and the relative transcript levels of Halloween gene *Cs-Phm* in the rice striped stem borer, *C. suppressalis*. It has been identified in several insects species that Halloween genes are involved in ecdysteroidogenesis [8,19,24,42]. Helix-C, Helix-I, Helix-K, PERF motif, and heme-binding domain are well-conserved typical cytochrome P450 motifs [43]. Previous studies have reported similar P450 motifs of Halloween genes in Lepidoptera, Diptera, Hemiptera, Hymenoptera, Orthoptera, and Coleoptera [20,35,36,37,39,42,44,45].

*Cs-Phm* has Helix-C, Helix-I, Helix-K, PERF, and heme-binding motifs. Consistent with the structural features, the similar P450 motifs in *Cs-Phm* are believed to have similar functions. Similarly sequences with an N-terminal part have microsomal enzyme character [34]. Moreover, phantom microsomal localization is also detected in phantom transfected Drosophila S2 cells [19]. *Cs-Phm* shows similar microsomal localization in sequence. This correlation strongly suggests that with the structural features and motifs, *Cs-Phm* is functionally conserved in the rice striped stem borer. Moreover, the spatial distribution of *Cs-Phm* supports our hypothesis that strong expression is limited in the prothoracic gland.

As in previous qRT-PCR and in situ hybridization studies, it has been described in different insects such as *Drosophila melanogaster*, *Manduca sexta*, *Schistocerca gregaria*, *Bombyx mori*, and *Spodoptera littoralis* that phantom Halloween genes in immature insect stages are highly expressed in the prothoracic gland cells [19,35,36]. The significantly high relative expression of *Cs-Phm* in the prothoracic gland strongly suggests that it is involved in ecdysteroids biosynthesis because it is a major site of ecdysone production.

Consistent with our results, Lepidoptera and Diptera insect species show similar relative expression [9,17,18,42,46]. Trace amounts of *Cs-Phm* transcripts were also found in other tissues besides PG, suggesting a possible additional role of this gene might in other physiological functions in insects [47,48,49]. Furthermore, the relative expression pattern of phantom coincides with circulating ecdysteroid quantity in the hemolymph [19]. Therefore, it can be speculated that the temporal expression pattern of phantom is related to the 20E titer in the hemolymph. The observed temporal transcript pattern of *Cs-Phm* in the rice striped stem borer reflects the repetitive surges of ecdysteroid biosynthesis that act to coordinate molting and metamorphosis. Consequently, it seemed reasonable to expect the functional importance of *Cs-Phm* for the biosynthesis of ecdysteroids.

To validate our hypothesis that *Cs-Phm* is involved in the biosynthesis of ecdysteroids, we downregulated it using RNAi to evaluate the possible effects on larval development. The target gene was effectively knocked down by injection of ds*Cs-Phm*. RNAi knockdown significantly downregulated the relative transcript level in the ds*Cs-Phm*-injected group which led to a significantly reduced ecdysteroid titer. Similar findings have also been reported of reduced ecdysteroid titers with the loss of Halloween enzymes [17,18,24,35,36,37,49,50]. We also noted that negative effects in immature stages, such as mortality and delayed larval development, were observed in ds*Cs-Phm*-injected insects because of the reduced ecdysteroid titer. Similar findings have been documented to those of lepidopteran species *Bombyx mori*, *C. suppressalis*, and *Plutella xylostella* that downregulated Halloween genes animals resulted in longer developmental duration, lower pupation, phenotypic defects as well as mortality [21,51,52]. Moreover, these findings are similar to those of Wan et al., for hemipteran insects *Laodelphax striatellus*, where mortality and delayed developmental effects were found in downregulated Halloween gene animals [53].

Consistent with our speculation, similar findings were observed in *Toxoneuron nigriceps* with the parasitization of *Heliothis virescens*, where downregulation of Halloween gene induces downregulation of ecdysone production [54]. We also confirmed that the negative effects of ds*Cs-Phm*-injected insects can be rescued by topical application of 20E. The observed insects were almost completely rescued by application of 20E, and negative effects such as delayed development were relieved.

## 5. Conclusions

The Halloween gene phantom is evolutionarily conserved in *C. suppressalis.* The spatio-temporal expression patterns and RNAi knockdown of *Cs-Phm* showed a significant correlation with the ecdysteroid titer. This suggests the importance of *Cs-Phm* in ecdysone biosynthesis by prothoracic glands. We expect that in future studies, knowledge of *Cs-Phm* will lead to practical application in managing this insect pest.

## Data Availability

The data presented in the study are available in the article.
